# Brain Aging and Gut–Brain Axis

**DOI:** 10.3390/nu11020424

**Published:** 2019-02-18

**Authors:** M. Hasan Mohajeri

**Affiliations:** Department of human medicine, University of Zurich, Winterthurerstrasse 190, 8057 Zürich, Switzerland; mhasan.mohajeri@uzh.ch; Tel.: +41-79-9381203

In the last decade, the microbiome in general and the gut microbiome in particular have been associated not only to brain development and function, but also to the pathophysiology of brain aging and to neurodegenerative disorders such as Alzheimer’s disease (AD), Parkinson’s disease (PD), depression, or multiple sclerosis (MS). Increased life expectancy in the 20th century, and the associated dramatic demographic shift, necessitate the search for effective measures to cope with the intensifying need for the diagnosis and the management of neurological and neurodegenerative disorders. A large body of literature provide compelling evidence for the existence of a direct bidirectional crosstalk between the brain and the gut, pinpointing to the fact that they have a reciprocal influence on each other’s physiology and (dys)function.

Identifying the possible parameters of these interactions may pave the way for a better understanding of the mechanistic paths of healthy brain functioning, and may hold the potential to help to develop causative treatments for the psychiatric and neurodegenerative illnesses. Consequently, tremendous efforts, combining preclinical and clinical investigations, have been carried out and are currently underway. Preclinical research has already identified a plethora of microorganisms that may impact brain development, maturation, aging, and functions. Some of the data have been confirmed in healthy or diseased human cohorts. It must be noted; however, that human association studies on their own are, at best, correlative, and placebo-controlled clinical trials in large cohorts of representative human populations are needed to assess the disease-modifying impact and to unequivocally establish causation. 

This special issue contains systematic reviews and original papers highlighting the roles of microbes and diets on the onset and physiology of brain-related disorders. 

Corpuz et al., show, in the senescence-accelerated mouse prone 8 (SAMP8) model of premature aging [1], that a long-term supplementation with *Lactobacillus paracasei* K71 led to a better cognitive performance. They provided a possible functional link by showing that the supplemented mice exhibited an enhanced serotonin level, an elevated expression of brain-derived neurotrophic factor (BDNF), and higher phosphorylation of cAMP response element binding protein (CREB) in the hippocampus. These changes are hallmarks of sustained neuronal plasticity [2,3,4,5,6], which is crucial for supporting the learning process. The authors suggest that daily intake of *Lactobacillus paracasei* K71 may be a promising preventative strategy for age-related cognitive decline in the elderly.

Paley and Perry illustrate a link between the dietary tryptophan metabolite tryptamine, which is produced by human gut microbiome and neurodegeneration *in vitro* and in mice. Tryptamine inhibits tryptophanyl-tRNA synthetase, a key enzyme of protein biosynthesis [7,8]. These data suggest that defective protein synthesis may play a causative role in neurodegeneration. Staphylococci produce tryptamine and are found in 60% of human stool samples [9]. The authors also show that microbial production of tryptamine can be modified by human diet, drugs, and dietary supplements. The presented data indicate that the inhibition of tryptophanyl-tRNA synthetase by tryptamine and other tryptophan metabolites may impair protein biosynthesis and may, consequently, cause neurodegeneration.

La Rosa and colleagues review clinical trials aiming to understand the role of the omega-3 fatty acids to modulate the gut–brain axis and AD symptoms. Indeed, the knowledge of the interactions between gut-associated immune, enteric, endocrine, and nervous systems and the brain [10] has long been under-represented when designing therapies for psychiatric and neurological diseases. Evidence is presented that docosahexaenoic acid (DHA) and eicosapentaenoic acid (EPA) supplementation increase the production of short chain fatty acids (SCFA), shifting gut microbiota composition towards an eubiotic condition. Therefore, omega-3 fatty acids may be considered prebiotics. These nutrients are; however, adsorbed in the intestine and normally would not reach the colon. In order to exert a full prebiotic activity, a targeted delivery strategy of the nutrients must be envisaged to deliver them directly to the microbiome of the colon. Randomized placebo-controlled double-blinded clinical trials will be necessary to clarify the effect of omega-3 fatty acid supplementation in well-selected AD patients.

In their review, Kujawska and Jodynis-Liebert examined the potential of polyphenols as therapeutic agents for managing PD, the second most common neurodegenerative disorder. On the mechanistic level, polyphenols have been convincingly shown to inhibit alpha-synuclein misfolding and activate cellular pathways involved in neuroprotection in cellular models. These data; however, cannot be easily extrapolated to intact animal bodies, as the action of polyphenols will depend on their biotransformation and the ability to penetrate the blood–brain barrier (BBB) [11]. Extensive lists of animal studies and human data regarding the relationship between polyphenol consumption and the incidence of neurodegenerative diseases is presented in this review, allowing the conclusion that polyphenols exert neuroprotective effects. Nonetheless, the knowledge of their mode of action is limited, as little is known as to whether they reach CNS in substantial amounts. Polyphenols undergo extensive metabolization after ingestion. Therefore, it is critical, for understanding their therapeutic potential, to study their metabolism and the bioavailability and brain distribution of their metabolites *in vivo*. New data suggest that gut microbiota may play a role in biotransformation and; thus, in the biological activity of high-molecular-weight polyphenols, and several polyphenols seem to favor the growth of specific bacteria in the colon [12]. Taken together, polyphenols have a potential to protect neurons from neurodegenerative processes in PD. It can be expected that additional research will identify metabolically active and bioavailable polyphenol metabolites with a realistic therapeutic potential against PD.

Lastly, we evaluated, in a systematic review, the available human data describing the changes in the microbiome composition in patients suffering from neurodegenerative disorders, including AD, PD, MS, multiple system atrophy (MSA), neuromyelitis optica (NMO), and amyotrophic lateral sclerosis (ALS), and compared them with each other and with those in healthy age-matched controls. We show that most changes are inconsistent at low taxonomic resolution (for example at phylum level), thus have no predictive validity [13]. The reason lies in the functional redundancy. Varying changes in abundances of families in one phylum may level out each other, such that no significant changes may be observed at the phylum level. In contrast, identifying the precise microbiome changes at species level may very well help distinguish between the neurodegenerative diseases, even before the clinical manifestation of these diseases. Such diagnostic change of the species is demonstrated for PD in our review article [13]. Here, we additionally call for mythological standardizations in microbiome research to ensure the comparability of data and a better chance of diagnosis of diseases. 

Given the need for developing sustainable and side effect free therapies against brain-related diseases [14], it is of upmost importance to research the potential diagnostic and therapeutic role of the microbiome in detail. Recent data show, doubtlessly, that the gut microbiome plays a role in the pathophysiology of many human diseases [15,16]. Therefore, research in the near future must provide certain evidence as to which changes in the microbiome will have diagnostic value, and whether such changes may be taken as a basis to design therapeutic approaches against neurological and neurodegenerative disorders. 
